# A Real-Time Orbit Determination Method for Smooth Transition from Optical Tracking to Laser Ranging of Debris

**DOI:** 10.3390/s16070962

**Published:** 2016-06-24

**Authors:** Bin Li, Jizhang Sang, Zhongping Zhang

**Affiliations:** 1 School of Geodesy and Geomatics, Wuhan University, Wuhan 430079, China; jzhsang@sgg.whu.edu.cn; 2 Collaborative Innovation Center for Geospatial Technology, Wuhan 430079, China; 3 Shanghai Astronomical Observatory, Chinese Academy of Science, Shanghai 200030, China; zzp@shao.ac.cn

**Keywords:** debris laser tracking, telescope pointing, angular data, orbit determination and prediction

## Abstract

A critical requirement to achieve high efficiency of debris laser tracking is to have sufficiently accurate orbit predictions (OP) in both the pointing direction (better than 20 arc seconds) and distance from the tracking station to the debris objects, with the former more important than the latter because of the narrow laser beam. When the two line element (TLE) is used to provide the orbit predictions, the resultant pointing errors are usually on the order of tens to hundreds of arc seconds. In practice, therefore, angular observations of debris objects are first collected using an optical tracking sensor, and then used to guide the laser beam pointing to the objects. The manual guidance may cause interrupts to the laser tracking, and consequently loss of valuable laser tracking data. This paper presents a real-time orbit determination (OD) and prediction method to realize smooth and efficient debris laser tracking. The method uses TLE-computed positions and angles over a short-arc of less than 2 min as observations in an OD process where simplified force models are considered. After the OD convergence, the OP is performed from the last observation epoch to the end of the tracking pass. Simulation and real tracking data processing results show that the pointing prediction errors are usually less than 10″, and the distance errors less than 100 m, therefore, the prediction accuracy is sufficient for the blind laser tracking.

## 1. Introduction

Provision of accurate position information of space debris objects is dependent on the availability of accurate tracking data. The debris laser ranging (DLR) technique [1,2,3,4] developed over the last decade is able to measure the distance between a ground tracking station and a low Earth orbiting (LEO) debris object with about 1 m accuracy. This accuracy is one order higher than that of either the optical or radar tracking, the two most popular debris tracking techniques. However, different from the satellite laser ranging (SLR) system, DLR, operating on the uncooperative debris objects, has two distinctive features, among others. The first is that, because debris objects do not have laser retro-reflectors onboard, a DLR system would need more laser power to ensure sufficient laser signals can be returned back from debris objects and received by laser detectors. The second is related to the orbit predictions (OP) accuracy of debris objects. Unlike the SLR system which uses accurate consolidated prediction format (CPF) [5] files generated by the International Laser Ranging Service (ILRS) [6] to perform laser ranging to SLR satellites, DLR operations are currently aided by optical tracking. Therefore, SLR can operate 24 h a day, but DLR is only possible during the terminator periods, at present. This dictates the need for a fast and smooth transition from the optical tracking to laser ranging of the debris to allow the DLR system to make use of the valuable operation time. It is clear that, among other requirements, the debris OP accuracy is critical to realize the day-time DLR operation.

As only TLEs of a debris object are available, the OP pointing errors from them are usually on the order of tens to hundreds of arc seconds. Considering the realistic scenario of current DLR operations, in which the laser beam is usually only a few arc seconds wide, optical tracking is needed first to collect angular data, and then the angular data is used either to improve the orbit prediction through a real-time orbit upgrade technique [7], or to manually guide telescope pointing [3]. When the latter approach is applied, the debris object should be optically visible through the whole pass, which would strongly affect the efficiency of DLR operations. A better way is to use angular data to generate sufficiently accurate orbit predictions, such that the DLR can be successfully operated for the remaining part of the pass. Accurate orbit predictions are also required for debris orbital maneuver using ground-based lasers [8,9] and reliable orbit conjunction assessments. Essentially, DLR operations require the optical tracking first, and then a transition from the optical tracking to the laser ranging.

This paper presents a real-time orbit determination (OD)/OP method to realize a smooth transition from optical tracking to efficient debris laser ranging. With the improved orbit predictions of the debris, telescope pointing toward the debris, and distance between the tracking station and the debris, are sufficiently accurate for the DLR operations. Therefore, there is no need for further optical tracking after the transition.

The method uses TLE-computed positions and angles, over a short-arc of less than 2 min from the start of a tracking pass, as observations in an OD process where simplified force models are considered. The use of TLE-computed positions as observations in an OD process was proposed by Levit and Marshall [10] and Bennett, et al. [11]. After the OD convergence, the OP is performed from the last observation epoch to the pass end. The method is experimented with simulation and real tracking data.

Within the existing DLR systems, it appears the Electro Optic Systems (EOS), an Australian technology company operating in the aerospace and defense markets, debris tracking system, located at Mt Stromlo, Canberra, uses a real-time orbit update algorithm to provide accurate OP for the DLR operations. Although details of the EOS algorithm are not published, it is understood that the algorithm uses the optical angular data to refine only some of the six orbit elements, which is different from the strategy adopted by the proposed algorithm presented in this paper.

In what follows, the method is presented in Section 2, followed by the experiment results in Section 3. Finally, some conclusions are given.

## 2. Methods

### 2.1. Real-Time Orbit Determination and Prediction Method

The motion of an Earth orbiting object is generally described by the following equation:

(1) $$\ddot{r}=f(r,\dot{r},c,t)$$

where r, $\dot{r}$, and $\ddot{r}$ are the position, velocity, and acceleration vectors of the object in an Earth-centered inertial coordinate system, c is the vector of force model parameters, and f is the vector of the force per unit mass exerted on the object.

For LEO space objects, the following forces are usually included in the orbit computation: the Earth’s gravity, solar-lunar and planetary gravities, air drag, solar radiation pressure, and the Earth tidal forces, including the solid Earth and ocean tides.

For many geodetic and geophysics applications, such as satellite altimetry measuring sea-level variations, orbit information of centimeter accuracy is required, and it can be provided through the precise orbit determination (POD) procedure in which accurate and densely-distributed observations are processed [12,13,14]. When the accuracy of the dense tracking observations, like GPS carrier phases [15] or SLR data [16] is in the order of millimeters, the resultant POD accuracy is in the order of centimeters [17,18].

For applications in the space situational awareness, people are generally more concerned with the OP accuracy of space debris. Although the OP of many debris objects can be computed using the publicly-available TLE with SGP4 algorithm [19], its accuracy should be improved in order to meet more advanced requirements; for example, accurate and reliable space collision warning. Such improvement would be achieved through either the use of more quality tracking data, or the refinement of perturbing force models, or both of them.

For the DLR operations, the purpose of the real-time OD using angular data over a short-arc is to generate OP from the last observation epoch to the end of pass, and that the generated OP is accurate enough for the DLR telescope pointing and distance between the tracking station and the debris. To make the transition from the optical tracking to the DLR as fast as possible, one would demand the OD use as little angular data of the object as possible. It is well known, however, when only angular data over a short orbit arc is available, the OD computation would not converge because of the poor distribution of tracking data and the unknown ballistic coefficient of the object [20,21,22]. To achieve the OD convergence, extra information has to be used in the OD computation. In the case of debris optical or laser tracking, the most likely available extra information is the TLE of the debris, which can be downloaded from the North American Aerospace Defense Command (NORAD) catalogue [23]. The TLEs are widely used to propagate the orbits of space objects to any moment with the SGP4 algorithm. Therefore, the TLE-computed positions can be used as extra information or pseudo-observations, in addition to the collected short-arc angular data, to achieve the OD convergence [24]. The TLE of a debris object may be updated each day. In practice, one usually uses the latest TLE to propagate orbits since the latest TLE is usually more accurate than earlier TLEs.

Given a set of TLEs, one can compute the position at any moment using the SGP4 algorithm. Considering that the angular data is only available over a short orbit arc, and the use of TLE-computed positions is mainly to achieve the OD convergence, one should not use more-than-necessary TLE-computed positions. The overuse of the TLE-computed positions would cause the OD results to be biased toward the inaccurate TLE orbit. Earlier experiences [23] have shown that the TLE-computed positions every 10 min within the OD span are sufficient to help achieve the OD convergence in case only the angular data over a short orbit arc is available.

The OD problem discussed in the paper can now be formulated as: for a debris object to be laser ranged, given its angular observations collected over a short-arc, and a set of its latest TLE prior to the tracking time, the OD is performed in a specified OD span in real-time using the angular data and TLE-computed positions as observations, and the subsequent OP is sufficiently accurate for blind DLR operations to the debris object.

When the OD span is set, the positions every 10 min are first computed from the latest TLE, and are used as pseudo-observations. Assume that *n* pseudo-observations, $O_{TLE-k},\;k=1,2,\cdots ,n$, are available, and the weight for each of these positional observations is $P_{TLE}$. Each position has three components in *X*, *Y*, and *Z*, and they are treated equally.

The other observations are the angles data of the objects, which can be in the form of azimuth and elevation, or topocentric right ascension and declination. Assume that *m* azimuth/elevation observations, $O_{AzEl-k},\;k=1,2,\cdots ,m$, are available, and the weight of each observation is $P_{AzEl}$. The relationship between the azimuth/elevation and the positions of the space object and ground tracking station can be found in [25].

With the above observations and their weights as input to an iterative least-squares (LS) OD program, the initial state vector (position and velocity vectors at the start of the OD span) of the object, as well as force model parameters, can be adjusted to minimize the observational residuals in the least-squares sense. That is, the weighted sum of squares of the residuals (WSSR) of the observations

(2) $$WSSR=\overset{n}{\underset{k=1}{\sum }}P_{TLE}v_{TLE-k}^{2}+\overset{m}{\underset{k=1}{\sum }}P_{AzEl}v_{AzEl-k}^{2}$$

is minimized, where the observational residuals are computed as:

(3) $$v_{TLE-k}=O_{TLE-k}-C_{TLE-k}$$

(4) $$v_{AzEl-k}=O_{AzEl-k}-C_{AzEl-k}$$

The computed value, C, of the corresponding observation is computed from the integrated positions of the object. In the OD program, the 11-order Cowell numerical orbit integration method is used for orbit propagation with an integration step size 30 s. The TLE of the object is used to compute the initial state vector for the OD process.

The OD process is considered converged when changes in the initial state vector and estimated force model parameters (if applicable) are less than preset values. After the OD convergence, an outlier detection procedure is applied to make sure no observation with gross error is used in the OD computation. In the outlier detection, TLE-computed positions and the angular observations are treated separately as two groups. For each group, the RMS of the residuals is computed first, and then each residual is checked against three times the RMS value. When the absolute value of a residual is larger than the three times the RMS value, the corresponding observation is marked. If there exists any outlier in the observations, the OD process is repeated in which the marked observations are not used. After the above OD process, the orbit is propagated until the end of the pass end, and the OP results are delivered to the DLR system.

**Figure 1:**
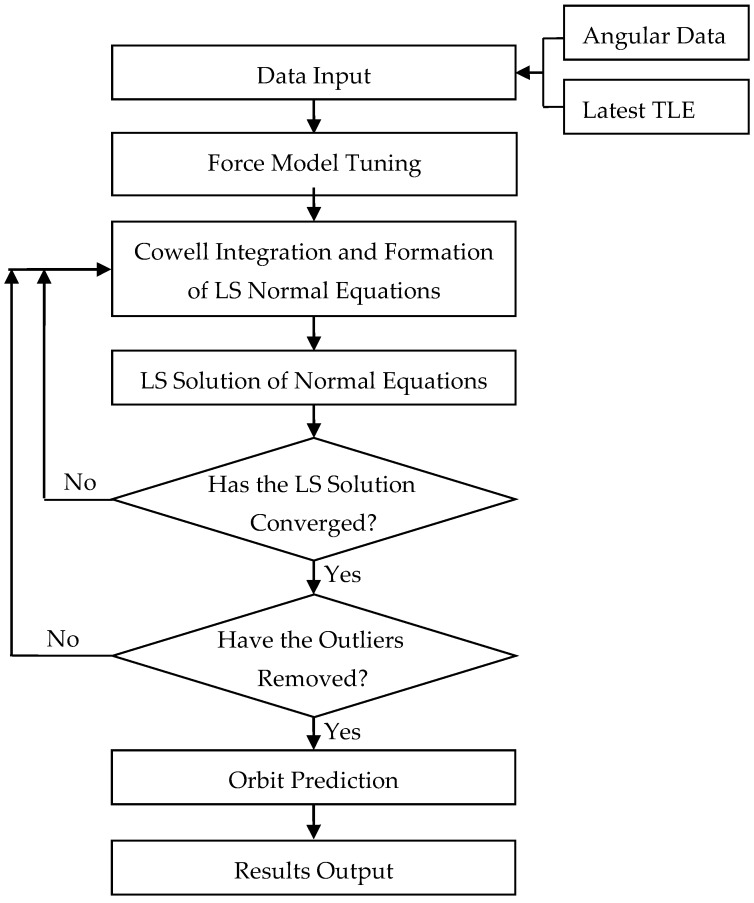
Structure of the real-time OD/OP program.

The flowchart of the OD/OP process using the TLE-computed positions and angular observations is shown in Figure 1.

The accuracy of the angular data is usually at the level of 2″–5″ [7], which is equivalent to 10–25 m at a distance of 1000 km. The accuracy of the TLE-computed positions is usually at hundreds of meters [10,26]. This raises the question on how to assign appropriate weights to the angular data and TLE-computed positions in the OD computation. As mentioned before, the use of TLE-computed positions is to help the OD process converge. Therefore, the TLE-computed positions should not be overused to avoid the OD results biased toward the TLE, and this is achieved by the adjustment of the weight assigned to the TLE-computed positions in the proposed algorithm. In the algorithm design, the OD span is fixed to 12 h (see next paragraph). This means the number of the TLE-computed positions used in the OD computation is fixed since the positions are computed every 10 min. On the other hand, the number of angular observations and the geometrical strength of the collective observations mainly depend on the length of the orbit arc over which the optical data is collected. Therefore, when a shorter arc, for example, 30 s, is optically tracked, the weight assigned to the TLE-computed positions should be smaller than that when a longer arc is optically tracked. Experiments have shown that when the optical tracking arc is 30 s, a weight of 10^−17^ for the TLE-computed positions is appropriate in term of the OP accuracy; while a weight of 10^−16^ is appropriate when the optical tracking arc is 60 s or longer. The weight for the angular data is set 1.0. Although there is still space to improve the OP accuracy by the adjust of the weight for the TLE-computed positions, the pursue for that improvement is not made because this paper is focused on whether the proposed algorithm can generate OP results with the accuracy required by the smooth transition from the optical tracking to laser ranging.

For the tracking purpose, the OD and OP computation has to be completed in real-time. This would require the OD span be short to save computation time but long enough to guarantee the OD convergence and accuracy. Experiments have shown that if the OD span is set starting 12 h before the tracking time and ending at the last observation epoch, both the OD convergence and accuracy are achieved.

In a short summary, when only the angular data over a short arc of less than 2 min is available, the TLE-computed positions at 10 min interval will be used as pseudo-observations in the OD computation, with a weight of 10^−16^–10^−17^. The OD span is about 12 h long, ending at the last observation epoch.

### 2.2. Force Model Consideration

When a large volume of data over a long time span is processed, the POD computation could take minutes to converge and, thus, is not suitable for real-time applications. A breakdown of the POD computation time reveals that most of the time is spent on the computation of the perturbing accelerations from the Earth’s gravity and the atmospheric drag.

Generally, the pass duration of a LEO debris object is less than ten minutes with respect to the tracking station, which means the OP time spans are short. In such scenarios, considering full perturbations in the OD and OP computations will not substantially contribute to the OD and accuracy improvement. Take one pass of GRACE-A satellite (more sensitive to the gravity and drag), for example; the whole pass duration with respect to the tracking station is 293.0 s. For the application scenario discussed in this paper, using 30 s angular data with the observation accuracy of 2″, the prediction errors (azimuth/elevation/distance) are 2.6″/3.3″/9.0 m, respectively, when only using a JGM-3 gravity model truncated to 20/20, while the angular errors are 2.6″/3.1″/8.5 m when the drag is included. It can be seen that the gain in the OP accuracy from considering the drag is minimal. Similarly, considering a gravity model with a higher degree/order in the discussed OD/OP scenario will only generate slightly better OP results. For example, when the full JGM-3 model of degree/order 70/70 is used, the prediction errors are 2.5″/3.1″/9.0 m, respectively for the azimuth, elevation, and distance. Other perturbation forces, such as the third-body gravities and solar radiation pressure, can be neglected for the discussed problem. Thus, it is only necessary to consider a gravity model truncated to degree/order of 20/20. That is, almost the same OP results for the time period from the last observation epoch to the pass end are obtained whether a complete set of force models or only the truncated gravity model is considered in the OD computation.

### 2.3. Software

An orbit determination and analysis software for Earth-orbiting objects has been developed at School of Geodesy and Geomatics, Wuhan University (China). The software has the following main functions: OD using various tracking data and considering various perturbing forces, orbit and tracking data simulations, atmospheric mass density calibration, and space conjunction analysis.

A tailor-made version for the application in this paper is developed. It simulates the debris optical-laser tracking scenario: given a set of TLE of a debris object, optical tracking is first performed to collect the angular data, and then the real-time OD using the collected angular data and TLE-computed positions is conducted, and, finally the OP results are fed to the laser tracking system to start the laser ranging operation. The OD process may be repeated to update the OP results when new angular observations are collected.

On the basis of above discussions, the settings for the software are summarized in Table 1. With these settings, the OD and OP computation is completed in less than 2 s, satisfying the real-time requirement for the application.

## 3. Experiment Results

Both simulation and real tracking data is processed using the tailor-made software. The performance is assessed with the root mean square (RMS) of the differences between the predictions and “truth” data, which are either the computed values from the “truth” orbit or the real tracking data.

### 3.1. Experiments with Simulation Data

Four geodetic satellites in the LEO region are chosen in the simulation data experiments: GRACE-A, Larets, Starlette, and Ajisai, whose perigee altitudes are 393 km, 690 km, 815 km, and 1479 km, respectively. At present, the DLR systems mainly operate at the LEO objects. The chosen four satellites are to represent debris objects at different altitudes in the LEO region. Their accurate CPF-format predictions are available on the ILRS data center CDDIS (ftp://cddis.gsfc.nasa.gov/slr/cpf_predicts/) [5]. For these experiments, the CPF files in the second half of 2014 of these satellites are downloaded. Only the orbit data on the first day in each CPF file is used because they are believed to be more accurate than those of later days.

In the simulation experiments, the CPF orbits are regarded as “truth” orbits and, thus, used to generate the angular observation data. The tracking station is set at Shanghai SLR station (SLR station ID: 7821). The angular data is output every 0.5 s, and observation accuracy of 2″ and 5″ is assumed, respectively.

For testing the effect of the arc length of the angular data on the OP accuracy, four OD cases using angular data over arc lengths of 120 s, 90 s, 60 s, and 30 s are considered. After the OD, the OP starting from the last observation epoch to the pass end is followed. This is to simulate the practical tracking scenario.

OD/OP simulations on 200 passes are performed for each of the two angular data accuracy assumptions and each satellite. Table 2 shows the statistics of the 200 pass durations of the four satellites with respect to Shanghai station.

**Figure 2:**
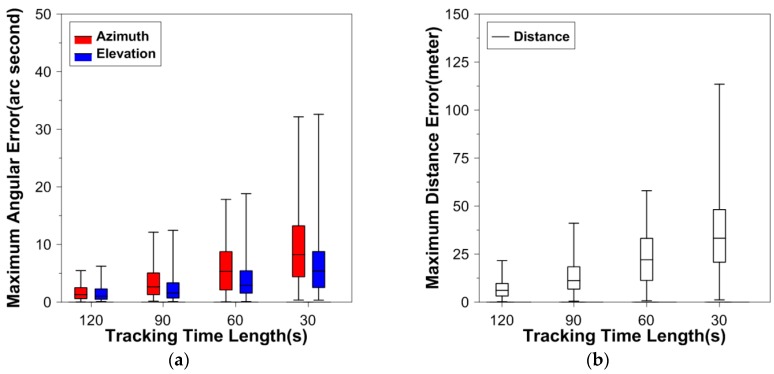
Boxplots of maximum prediction errors from the 200 computations for GRACE-A when the angular data accuracy is 2″. (**a**) describes the boxplots of maximum prediction errors for the azimuth (red) and elevation (blue); (**b**) describes the boxplots of maximum prediction errors for the distance (white).

**Figure 3:**
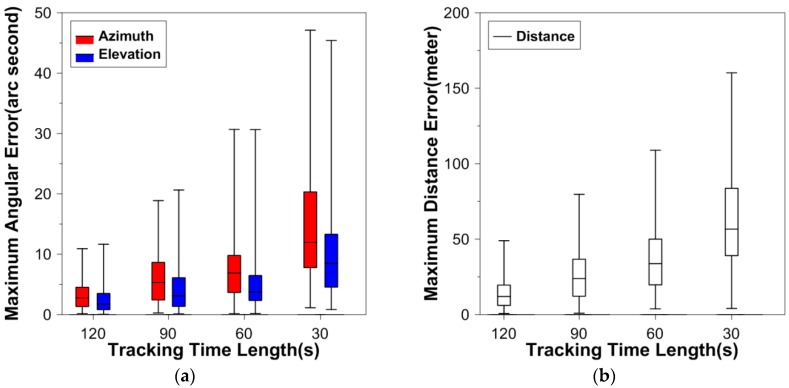
Boxplots of maximum prediction errors from the 200 computations for GRACE-A when the angular data accuracy is 5″. (**a**) describes the boxplots of maximum prediction errors for the azimuth (red) and elevation (blue); (**b**) describes the boxplots of maximum prediction errors for the distance (white).

Practices have shown that DLR to a lower altitude object is more difficult than to a higher object, because the rapid change in the azimuth and elevation demands more accurate telescope pointing control, which has strong dependence on the maximum prediction error. Among the four experiment satellites, the orbit altitude of GRACE-A is the lowest, which means the pass duration is relatively short (the motion of the satellite with respect to the tracking station is relatively fast). Figure 2 shows the boxplots of absolute maximum prediction errors from the 200 runs for GRACE-A when the angular data accuracy is 2″, and Figure 3 is for the 5″ accuracy.

From Figure 2, in the case of 2″ angular data accuracy, when the angular data arc length is 120 s, all of the 200 maximum angular (azimuth and elevation) prediction errors are less than 10″; when the arc length is 60 s, more than 75% of the angular prediction errors are less than 10″. As expected, when the arc length is only 30 s, the OP errors are larger compared to those of longer data arcs. In this situation, more than 50% of the maximum azimuth errors are larger than 10″. For the distance, all the maximum errors are less than 150 m. As for the angular data at the accuracy of 5″, the prediction errors are larger than those when the angular data accuracy is 2″, but the trend that the error increases with the decrease of the data arc is similar in both accuracy cases.

Regarding the occurrence times of the maximum prediction errors in the 200 passes, more than 50% of them occur in the last fifth of the OP span, and all of them occur in the second half of the OP span.

It is clear that the maximum prediction errors decrease when the angular data arc length increases. This fact is also shown in the RMSs of the prediction errors. Table 3 gives the average RMS values of prediction errors from the 200 computations for GRACE-A. Table 4, Table 5 and Table 6 are for Larets, Starlette, and Ajisai, respectively. In the tables, the average RMS values of the TLE/SGP4-predicted positions errors are also given in order to make a comparison.

From the tables above, it is seen that the TLE/SGP4 prediction accuracy does not meet the requirement for the blind DLR tracking.

When the real-time OD/OP is performed, even for GRACE-A at the low altitude of 393 km, angular observations over 30 s short-arc, whether the data accuracy is 2″ or 5″, are enough to generate OP results for the blind DLR tracking, with its angular prediction accuracy less than 10″ and distance prediction accuracy less than 100 m.

Observing the above simulation results, the OP accuracy for GRACE-A is clearly better than those for other three higher satellite. It is believed to be mainly due to the short OP span for GRACE-A. For the other three satellites, the angular prediction accuracy is generally at the same level, and the distance prediction accuracy of Starlette and Ajisai is slightly worse. There are possibly two factors: one is that the OP span for Starlette and Ajisai are longer than that for Larets, which would make the OP accuracy worse for the longer OP span. On the other hand, the accuracy of the TLE-computed positions for Starlette and Ajisai is significantly better than that for Larets, the contribution to the OD/OP from the TLE-computed positions is certainly more for Starlette and Ajisai. These two factors have opposite effects on the OP accuracy and, thus, may result in the generally same level OP accuracy.

The computation time for each OD/OP run of all the simulations is less than 2 s, which means the algorithm is implementable in real-time.

### 3.2. Experiments with Real Tracking Data

The above algorithm is tested to real tracking data. The real tracking data contains both the angular and laser ranging data. Three debris objects (NORAD ID: 26703, 1430, 6275) are processed to examine the effectiveness of the proposed algorithm. In the assessments, the real angular and DLR data is used as “truth” to compute the prediction errors.

#### 3.2.1. OP Errors for NORAD ID 26703

**Figure 4:**
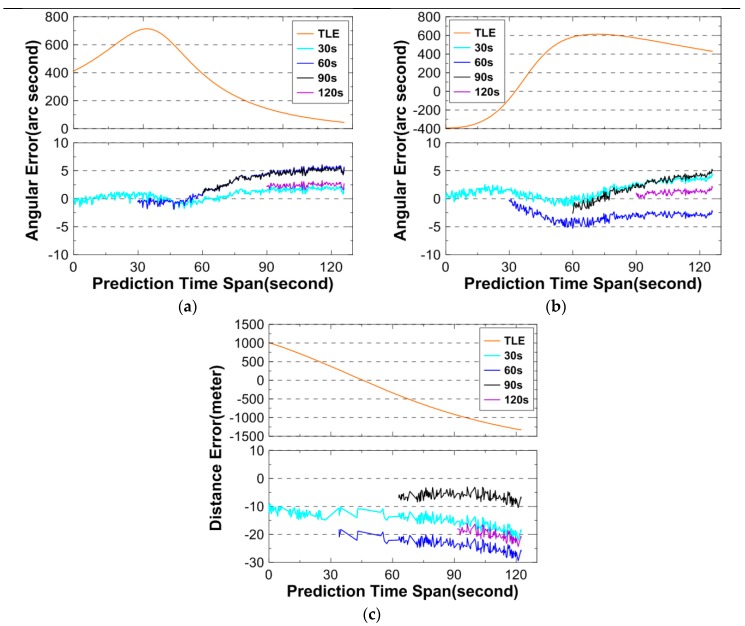
Angular and distance prediction errors for 26703. (**a**) describes the prediction errors for the azimuth; (**b**) describes the prediction errors for the elevation; and (**c**) describes the prediction errors for the distance. Above: prediction errors using TLE only; below: prediction errors using angular data and TLE.

The perigee and apogee altitude for 26703 are 585 km and 592 km, respectively. Table 7 gives basic information of the angular and DLR observations, and Figure 4 shows the prediction errors. In the figure, the TLE/SGP4 prediction errors are also shown.

Observing Figure 4, the maximum angular and distance errors are 5″ and 30 m, respectively, for all OD cases using angular data over 120 s, 90 s, 60 s, or 30 s arc. As is seen from the figure, the angular prediction errors using TLE are on the order of hundreds of arc seconds. The figure clearly shows the effectiveness of the algorithm.

#### 3.2.2. OP Errors for NORAD ID 1430

**Figure 5:**
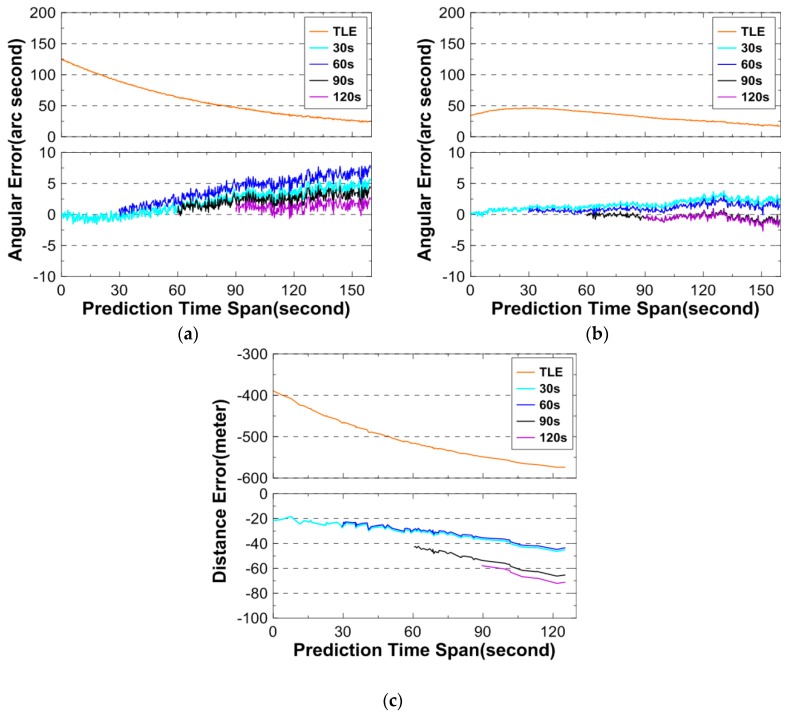
Angular and distance prediction errors for 1430. (**a**) describes the prediction errors for the azimuth; (**b**) describes the prediction errors for the elevation; and (**c**) describes the prediction errors for the distance. Above: prediction errors using TLE only; below: prediction errors using angular data and TLE.

The second test object’s NORAD ID is 1430, and it has perigee and apogee altitude at 718 km and 800 km, respectively. Table 8 lists basic information of the angular and DLR observations, and Figure 5 shows the prediction errors. It is noted that the number of the DLR points for this objects is significantly less than those for the objects of NORAD IDs 26703 (Section 3.2.1) and 6275 (Section 3.2.3) and the reason is not clear, although the setting of the DLR system is exactly the same for all three objects.

In Figure 5, the maximum angular prediction error is less than 10″ for azimuth and 5″ for elevation, respectively. The distance prediction error is within 80 m. The OP errors from using TLE are reduced significantly with the real-time OD/OP approach.

#### 3.2.3. OP Errors for NORAD ID 6275

**Figure 6:**
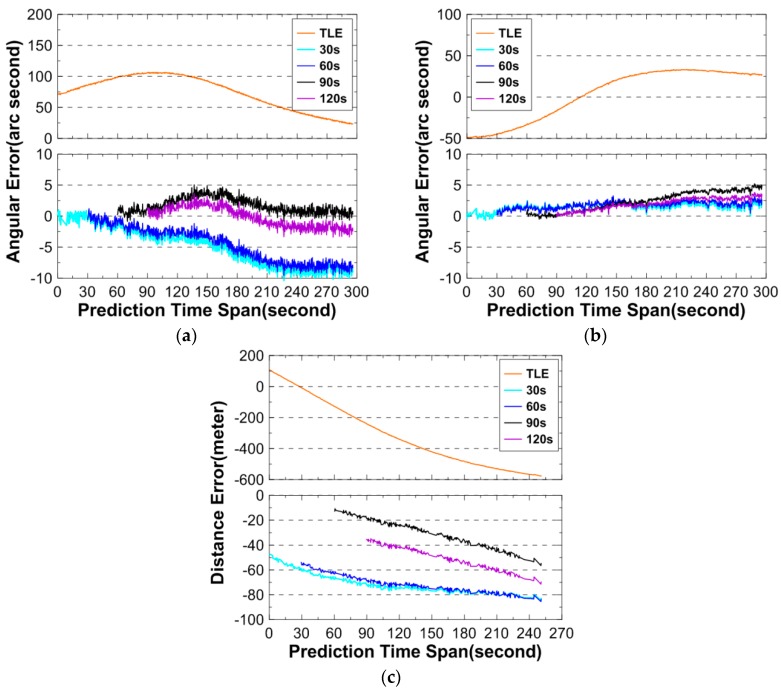
Angular and distance prediction errors for 6275. (**a**) describes the prediction errors for the azimuth; (**b**) describes the prediction errors for the elevation; and (**c**) describes the prediction errors for the distance. Above: prediction errors using TLE only; below: prediction errors using angular data and TLE.

The perigee and apogee altitude for 6275 are 776 km and 827 km, respectively. Table 9 gives the basic information of the angular and DLR observations for 6275, and Figure 6 shows the prediction errors.

The angular prediction errors for 6275 are within 10″ for azimuth and 5″ for elevation, and the distance prediction errors are within 90 m. Again, the OP errors from using TLE are reduced significantly with the real-time OD/OP approach.

### 3.3. Computation Time

The proposed algorithm is implemented using C++ in the Microsoft VS2010 environment running on a DELL computer (Intel (R) Core(TM) CPU 3.2 GHz). After receiving the angular observations, the software completes the OD/OP computations in less than 2 s for both the simulation and real tracking data, satisfying the real-time requirement of applying the proposed algorithm.

## 4. Conclusions

Debris laser ranging plays an important role in advancing the space debris environment management, particularly in the generation of precise orbit information. One requirement to ensure the success of the DLR operations is the accurate orbit prediction, such that the telescope can pinpoint to the debris object. At present, the optical tracking system is first applied to guide the narrow laser beam pointing to the objects, and this manual operation could interrupt the continuity of laser tracking, leading to the loss of valuable laser tracking data.

In this paper, a new real-time OD/OP algorithm for a smooth transition from optical to laser tracking has been developed. The method uses the short-arc angular observations and TLE-computed positions as pseudo-observations to determine the orbit of the space object with a simplified force model, and then to predict forward from the last observation epoch to the pass end. The method is tested with both simulation and real tracking data. The simulation results show that, for the LEO space objects, the OD using angular observations of 30 s short-arc can generate OP results in an accuracy of 10″ for angles and 100 m for distances, respectively, which can meet the requirement for the blind DLR operations. This conclusion is verified by the processing of real angular and DLR tracking data. Meanwhile, the OD and OP computation can be completed within 2 s, satisfying the real-time requirement for the practical application.

## Figures and Tables

**Table 1:** Models and Parameters Setting of the Software.

Model/Parameters	Setting
OD span	12 h ending at the last angular observation epoch
OP span	From the last observation epoch to the pass end
Observations	
Angular data	Weight: 1.0
TLE-computed positions	Interval: 10 min, weight: 10^−16^ –10^−17^
Forces	
Earth gravity model	JGM-3 truncated to degree/order 20/20
Others	Neglected
State vector	Position and velocity vectors at the begin of OD span
Reference frame	
Coordinate system	True of Date at the start of OD span
Precession and nutation	IAU1976/IAU 1980 simplified model
Earth orientation	IERS Bulletin A

**Table 2:** Statistics of satellite pass durations at Shanghai SLR station.

Satellite	Longest Duration (s)	Shortest Duration (s)	Average Duration (s)
GRACE-A	319.5	121.5	258.0
Larets	468.5	133.5	363.5
Starlette	738.5	152.5	498.0
Ajisai	957.5	228.5	745.6

**Table 3:** Average RMSs of the prediction errors from 200 computations for GRACE-A.

Accuracy Arc Length	2″				5″				TLE
	120 s	90 s	60 s	30 s	120 s	90 s	60 s	30 s	
Azimuth (″)	1.2	2.3	3.6	5.8	2.2	4.1	4.5	9.3	237.1
Elevation (″)	1.1	1.7	2.7	3.9	1.9	2.9	3.2	6.0	94.0
Distance (m)	5.5	10.8	17.7	27.7	11.4	21.6	29.0	47.5	610.3

**Table 4:** Average RMSs of the prediction errors from 200 computations for Larets.

Accuracy Arc Length	2″				5″				TLE
	120 s	90 s	60 s	30 s	120 s	90 s	60 s	30 s	
Azimuth (″)	2.4	4.3	5.6	6.4	3.9	4.9	6.1	10.4	113.5
Elevation (″)	1.9	2.6	3.2	4.8	2.8	3.1	4.2	8.2	54.6
Distance (m)	18.7	34.7	42.8	48.3	31.6	39.3	43.8	86.9	432.5

**Table 5:** Average RMSs of the prediction errors from 200 computations for Starlette.

Accuracy Arc Length	2″				5″				TLE
	120 s	90 s	60 s	30 s	120 s	90 s	60 s	30 s	
Azimuth (″)	2.8	3.3	4.0	5.7	4.8	5.6	8.0	9.7	29.0
Elevation (″)	2.1	2.4	2.8	4.4	3.7	4.4	5.8	6.5	18.2
Distance (m)	27.6	30.6	41.7	72.7	51.6	58.4	76.2	88.2	185.5

**Table 6:** Average RMSs of the prediction errors from 200 computations for Ajisai.

Accuracy Arc Length	2″				5″				TLE
	120 s	90 s	60 s	30 s	120 s	90 s	60 s	30 s	
Azimuth (″)	2.2	2.8	4.5	5.5	3.8	4.8	6.4	7.4	20.6
Elevation (″)	1.8	2.3	3.5	4.0	3.0	3.8	5.0	5.6	13.1
Distance (m)	27.7	32.2	53.5	63.0	46.0	56.3	81.7	82.9	145.1

**Table 7:** The angular and DLR observations for NORAD 26703.

	Pass Start (hh:mm:ss)	Pass End (hh:mm:ss)	Pass Duration (s)	Number of Data Points
Angular	8:32:16.86	8:34:53.19	156.3 s	516
DLR	8:32:20.72	8:34:53.14	152.4 s	567

**Table 8:** The angular and DLR observations for NORAD 1430.

	Pass Start (hh:mm:ss)	Pass End (hh:mm:ss)	Pass Duration (s)	Number of Data Points
Angular	9:46:22.23	9:49:31.87	189.6 s	628
DLR	9:46:45.91	9:49:21.86	155.9 s	115

**Table 9:** The angular and DLR observations for NORAD 6275.

	Pass Start (hh:mm:ss)	Pass End (hh:mm:ss)	Pass Duration (s)	Number of Data Points
Angular	9:29:53.10	9:35:19.63	326.5 s	1090
DLR	9:30:36.55	9:35:18.03	281.5 s	843
